# Ibrutinib for rituximab-refractory Waldenström macro-globulinemia

**DOI:** 10.18632/oncotarget.23400

**Published:** 2017-12-18

**Authors:** Maria Gavriatopoulou, Efstathios Kastritis, Meletios Athanasios Dimopoulos

**Affiliations:** Department of Therapeutics, Alexandra General Hospital, National and Kapodistrian University of Athens, School of Medicine, Athens, Greece

**Keywords:** Waldenström Macroglobulinemia, ibrutinib, chemoimmunotherapy, rituximab-refractory, treatment strategies

Waldenström Macroglobulinemia (WM) is a rare B-cell malignancy which is characterized by the bone marrow infiltration by monoclonal lymphoplasmacytic cells and the secretion of the monoclonal IgM paraprotein [1]. Chemoimmunotherapy based on rituximab is the standard treatment for patients with WM across all the phases of the disease. However, and despite the advances in the treatment options, the disease eventually becomes resistant, even to rituximab, and therefore novel treatments are currently needed. Whole-genome sequencing revealed a single activating somatic mutation in *MYD88* (MYD88^L265P^) in patients with WM [2] that activates nuclear factor κB (NF-κB) through two distinct pathways involving Bruton’s tyrosine kinase (BTK) and the interleukin-1 receptor–associated kinases (IRAK1 and IRAK4) [3]. Ibrutinib is an oral small molecule inhibitor which binds to BTK selectively and irreversibly, triggering apoptosis of WM cells harboring *MYD88^L265P^* mutation. The exceptional clinical activity observed in WM patients who were included in a phase 1 study provided the rationale for further evaluation in larger studies [3–4]. Treon et al investigated the role and the clinical utility of ibrutinib in a prospective, phase 2, open-label, multicenter trial of 63 patients with relapsed or refractory WM [5–6]. During the initial data analysis, 62% of the patients responded to ibrutinib therapy and based on these results on January 29, 2015, the FDA approved a new indication for ibrutinib (Imbruvica; Pharmacyclics) for the treatment of patients with WM. Thus, ibrutinib became the first drug to receive approval for the treatment of this disease [7]. In the era of widespread rituximab use for WM, the need for new therapeutic options for patients with rituximab-refractory disease is imperative. Therefore, Dimopoulos et al assessed the efficacy and safety of ibrutinib in patients with rituximab-refractory disease and the results of the iNNOVATE study were recently published in the Lancet Oncology. The activity of ibrutinib in this heavily pretreated and refractory to rituximab patients was remarkable providing durable responses [8]. The study enrolled 31 patients requiring treatment that received oral ibrutinib 420 mg once daily until progression or unacceptable toxicity. Median age was 67 years and 13 (42%) of 31 patients had high risk disease per the International Prognostic Scoring System for Waldenström Macroglobulinemia. All patients were rituximab-refractory, while the median number of previous therapies was four. At 18 months overall response rate was 90%, with a median time to first response of 1 month, the estimated 18-month progression free survival was 86% and the estimated 18-month overall survival rate was 97%. The toxicity of ibrutinib was manageable and only two patients discontinued treatment due to adverse events. In this study, the proportion of rituximab-refractory patients that responded was similar to what has been observed in patients who were not refractory to rituximab [5–6]. Thus, ibrutinib probably overcomes the adverse prognosis of rituximab resistance. Furthermore, ibrutinib achieved impressive results with regards to progression free survival and overall survival. Ibrutinib activity does not seem to be affected by well-defined prognostic features such as age, β-2 microglobulin, serum IgM levels, anemia and thrombocytopenia. There is evidence that *MYD88* and *CXCR4* mutational status can predict response to ibrutinib. However, the results must be interpreted cautiously due to the small number of patients evaluated so far. In the iNNOVATE study the presence of CXCR4 mutation was associated with slower responses but response rates were still very high. Ibrutinib seems to be more effective in less heavily pretreated patients; progression-free survival for patients with one or two previous lines of treatment was significantly better than in more heavily pretreated patients. Therefore, the role of ibrutinib in earlier lines of therapy and in previously untreated patients is currently under investigation in the randomized arms of the study. Yet, several issues such as the financial burden and the indefinite administration until unacceptable toxicity or progressive disease should be considered, especially compared to chemoimmunotherapy which is less expensive and is completed in a relatively short and definite time period. This study represents one of the largest efforts to establish a standard of care for the rituximab refractory patients with WM, therefore, can be used as a benchmark for the development of future clinical trials. So, should single-agent ibrutinib become the standard therapy for patients with relapsed WM refractory to rituximab? The answer is probably yes, given that current combination therapies provide lower responses rates and shorter PFS compared with single-agent ibrutinib. As we continue to investigate the best treatment options for patients with WM, one fact remains clear: the sustained responses and prolonged progression-free survival, combined with the manageable toxicity observed with ibrutinib monotherapy indicate that this chemotherapy-free therapeutic approach is a new treatment choice for patients with heavily pretreated rituximab-refractory Waldenström macroglobulinemia.
